# Supplementary material for: N-acetyltransferase Gene Variants Involved in Pediatric Idiosyncratic Drug-Induced Liver Injury
Source: Biomedicines. 2024 Jun 11;12(6):1288. doi: 10.3390/biomedicines12061288 (PMC11201799; doi:10.3390/biomedicines12061288)
Supplement: Supplementary file 1 [file biomedicines-12-01288-s001.zip › File S1-Table S1 NAT2.pdf]

- Table S1: Characteristics of 43 children with DILI: sex, age, race, diagnosis, drug treatment and RUMCAM score.

SEX	AGE (years)	RACE	DIAGNOSIS	DRUG	RUCAM SCORE
Male	5	Caucasian	Fever	Ibuprofen	9
Female	1	Caucasian	Pneumococcal meningitis	Rifampicin	7
Female	2	Caucasian	Systemic inflammatory response syndrome	Meropenem	7
Female	2	Caucasian	Sepsis	Meropenem	6
Female	3	Caucasian	Reunatoid arthritis	Methotrexate	5
Male	1	Caucasian	Tachyarrhythmia	Amiodarone	8
Male	1	Caucasian	Cardiomyopathy	Propranolol	7
Male	0,5	Caucasian	Sepsis	Meropenem	6
Male	1	Caucasian	Sepsis	Meropenem	4
Female	1	Caucasian	Pneumonia (Candida)	Voriconazole	7
Female	3	Caucasian	Seizures	Zonisamide	8
Female	3	Caucasian	Sepsis (Candida)	Voriconazole	6
Male	5	Caucasian	Sepsis (S.aureus)	Cloxacillin	7
Female	10	Caucasian	Tonsillitis	Amoxicillin	7
Male	15	Caucasian	Ataxia-teleangiectasia	Cotrimoxazole	6
Female	14	Caucasian	Encephalitis	Acyclovir	5
Female	5	Caucasian	Pulmonary tuberculosis	Isoniacid	8
Female	3	Caucasian	Hip synovitis	Ibuprofen	6
Female	3	Caucasian	Hip synovitis	Ibuprofen	5
Female	1,5	Caucasian	Juvenile idiopathic arthritis	Methotrexate	4
Male	5	Caucasian	Respiratory infection	Amoxicillin-clavulanic acid	6
Male	9	Caucasian	Bronchitis	Amoxicillin-clavulanic acid	7
Female	1	Caucasian	Respiratory infection	Amoxicillin-clavulanic acid	5
Male	0,5	Caucasian	Respiratory infection	Amoxicillin-clavulanic acid	4
Male	1	Caucasian	Pultaceous tonsillitis	Amoxicillin-clavulanic acid	5
Male	14	Caucasian	Tonsillitis	Amoxicillin-clavulanic acid	7
Male	5	Caucasian	Mastoiditis	Amoxicillin-clavulanic acid	6
Male	0,5	Caucasian	Respiratory infection	Amoxicillin-clavulanic acid	5
Male	10	Caucasian	Respiratory infection	Amoxicillin-clavulanic acid	5
Male	3,5	Caucasian	Vomiting	Green anise	6
Male	4	Caucasian	Vomiting	Green anise	8
Male	0,2	Caucasian	Aerocolia	Horsetail (Equisetum)	4
Male	5	Caucasian	Acute lymphoblastic leukemia	Methotrexate	8
Male	4	Caucasian	Acute lymphoblastic leukemia	Methotrexate	10
Male	3	Caucasian	Acute lymphoblastic leukemia	Mercaptopurine	11
Female	3	Caucasian	Acute lymphoblastic leukemia	Mercaptopurine	12

Female	4	Caucasian	Acute lymphoblastic leukemia	Methotrexate	8
Female	5	Caucasian	Acute lymphoblastic leukemia	Mercaptopurine	12
Male	5	Caucasian	Acute lymphoblastic leukemia	Methotrexate	11
Female	3	Caucasian	Acute lymphoblastic leukemia	Mercaptopurine	11
Female	12	Caucasian	Acute lymphoblastic leukemia	Methotrexate	11
Male	6	Caucasian	Acute lymphoblastic leukemia	Methotrexate	8
Male	5	Caucasian	Acute lymphoblastic leukemia	Mercaptopurine	12
