# Supplementary material for: N-acetyltransferase Gene Variants Involved in Pediatric Idiosyncratic Drug-Induced Liver Injury
Source: Biomedicines. 2024 Jun 11;12(6):1288. doi: 10.3390/biomedicines12061288 (PMC11201799; doi:10.3390/biomedicines12061288)
Supplement: Supplementary file 1 [file biomedicines-12-01288-s001.zip › File S2 Supplementary Material.pdf]

Protein-protein interaction and biological enrichment network

The free online tool STRING v12.0 (https://string-db.org/) was used to perform the protein-protein interaction network. This database provides the integration of published data on protein-protein interactions (both physical and functional associations).

Variants identified in our samples that were not annotated as synonymous and those variants that included annotations such as "pathogenic", "drug response" or "uncertain significance" were included in this analysis for the generation of the protein-protein interaction network.

According to the previously mentioned parameters, 4,871 variants were identified. Once the duplicate variants were discarded, a total of 897 variants were identified. These variants were entered into the STRING tool and 889 proteins with 7,221 interactions (Fig. 1A) were found in its database and used to generate the protein-protein interaction network (medium confidence (0.400)). The nodes that remained without interaction with other nodes were eliminated.

A k-means clustering was then performed for a total of 8 clusters, which were studied in depth to determine the cluster that could be most related to the target pathology of this study. The result was a cluster composed of 80 proteins and 416 interactions (Fig 1B). This cluster was extracted from the network to perform an independent biological enrichment analysis.

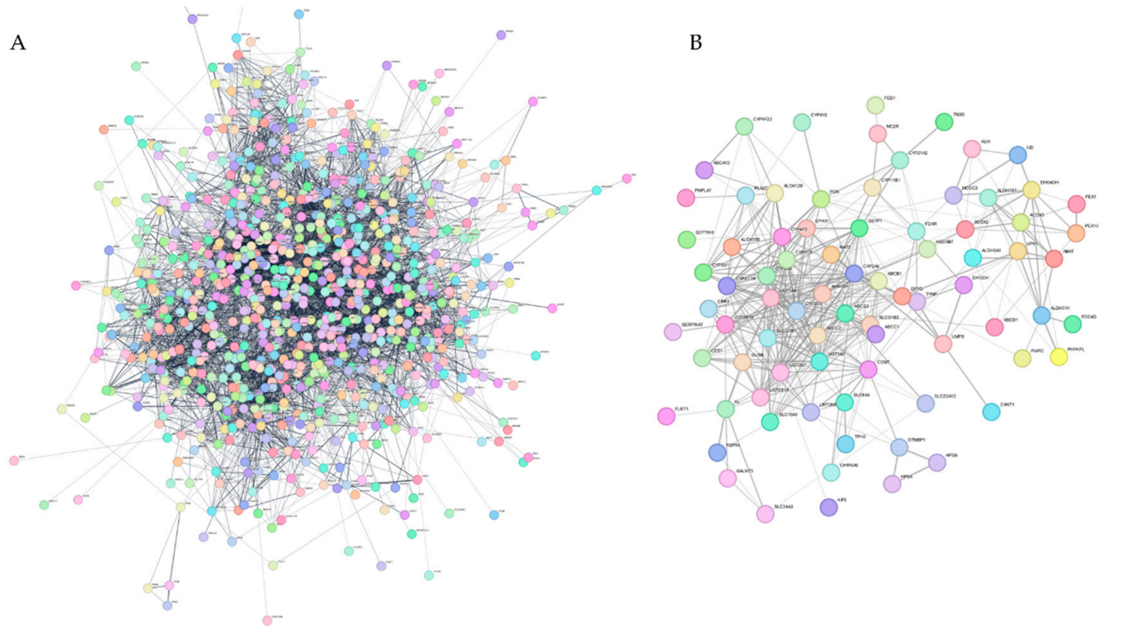

Figure 1. Protein-protein interaction networks of variants identified in pediatric patients with DILI. A: Protein-protein interaction network of all variants. B: Protein-protein interaction network of cluster 4 related to liver metabolism.

For the biological enrichment analysis of this cluster, the enrichment tools of the STRING database were used. Table 1 of this supplemental material shows the biological enrichment most closely related to DILI based on functions, biological processes, biological pathways or phenotype from the databases that STRING queries.

Table 1. Biological enrichment of the cluster of interest of variants identified from pediatric patients diagnosed with DILI.

**Category**	**Identifier**	**Description**	**False discovery rate**
GO Process	GO:0120188	Regulation of bile acid secretion	0.0318
GO Process	GO:0016098	Monoterpenoid metabolic process	0.0408
GO Process	GO:0043651	Linoleic acid metabolic process	0.00055
GO Process	GO:0006805	Xenobiotic metabolic process	1.97×10^{-16}
GO Process	GO:0009410	Response to xenobiotic stimulus	4.07×10^{-15}
GO Process	GO:0044255	Cellular lipid metabolic process	2.50×10^{-12}
GO Process	GO:0044238	Primary metabolic process	4.95×10^{-05}
GO Process	GO:0055085	Transmembrane transport	0.0225
GO Function	GO:0052869	Arachidonic acid omega-hydroxylase activity	0.0125
GO Function	GO:0022857	Transmembrane transporter activity	0.0447
GO Function	GO:0016491	Oxidoreductase activity	1.45×10^{-18}
GO Component	GO:0005737	Cytoplasm	0.00092
GO Component	GO:0016020	Membrane	0.0221
GO Component	GO:0016021	Integral component of membrane	0.0093
KEGG	hsa00983	Drug metabolism - other enzymes	8.08×10^{-17}
KEGG	hsa00120	Primary bile acid biosynthesis	0.0011
KEGG	hsa00982	Drug metabolism - cytochrome P450	6.20×10^{-13}
KEGG	hsa04976	Bile secretion	5.39×10^{-13}
KEGG	hsa00071	Fatty acid degradation	0.00057
KEGG	hsa01100	Metabolic pathways	6.06×10^{-30}
KEGG	hsa02010	ABC transporters	1.26×10^{-06}
Reactome	HSA-9749641	Aspirin ADME	3.44×10^{-12}

Reactome	HSA-9753281	Paracetamol ADME	6.99×10^{-07}
Reactome	HSA-9754706	Atorvastatin ADME	1.79×10^{-05}
Reactome	HSA-9748784	Drug ADME	4.77×10^{-19}
Reactome	HSA-194068	Bile acid and bile salt metabolism	7.15×10^{-06}
Reactome	HSA-5668914	Diseases of metabolism	1.09×10^{-05}
Reactome	HSA-8957322	Metabolism of steroids	2.51×10^{-06}
Reactome	HSA-556833	Metabolism of lipids	5.98×10^{-12}
Diseases	DOID:0014667	Disease of metabolism	4.29×10^{-16}
Diseases	DOID:655	Inherited metabolic disorder	9.60×10^{-15}
Diseases	DOID:1701	Steroid inherited metabolic disorder	9.42×10^{-06}
Human Phenotype	HP:0006566	Increased circulating ACTH level	0.0105
Human Phenotype	EFO:0004582	Liver enzyme measurement	0.0278
Human Phenotype	HP:0001392	Abnormality of the liver	0.0415
Human Phenotype	EFO:0004529	Lipid measurement	3.48×10^{-06}
Human Phenotype	EFO:0004732	Lipoprotein measurement	0.00050
Human Phenotype	EFO:0004725	Metabolite measurement	4.01×10^{-08}

GO: Gene Ontology; KEGG: Kyoto Encyclopedia of Genes and Genomes
